# Bronchiectasis in African children: Challenges and barriers to care

**DOI:** 10.3389/fped.2022.954608

**Published:** 2022-07-25

**Authors:** Charl Verwey, Diane M. Gray, Ziyaad Dangor, Rashida A. Ferrand, Adaeze C. Ayuk, Diana Marangu, Sandra Kwarteng Owusu, Muntanga K. Mapani, Ameena Goga, Refiloe Masekela

**Affiliations:** ^1^Department of Paediatrics and Child Health, Faculty of Health Sciences, University of the Witwatersrand, Johannesburg, South Africa; ^2^Research Council Vaccines and Infectious Diseases Analytics Research Unit, University of the Witwatersrand, Johannesburg, South Africa; ^3^Department of Paediatrics and Child Health, Red Cross Warm Memorial Children's Hospital and MRC Unit on Child and Adolescent Health, University of Cape Town, Cape Town, South Africa; ^4^Department of Clinical Research, London School of Hygiene and Tropical Medicine, London, United Kingdom; ^5^The Health Research Unit Zimbabwe, Biomedical Research and Training Institute, Harare, Zimbabwe; ^6^Department of Pediatrics, College of Medicine, University of Nigeria Teaching Hospital, Enugu, Nigeria; ^7^Department of Paediatrics and Child Health, University of Nairobi, Nairobi, Kenya; ^8^Department of Child Health, School of Medicine and Dentistry, Komfo Anokje Teaching Hospital, Kwane Nkrumah University of Science and Technology, Kumasi, Ghana; ^9^Levy Mwanawasa University Teaching Hospital, Lusaka, Zambia; ^10^HIV and Other Infectious Diseases Research Unit, South African Medical Research Council, Johannesburg, South Africa; ^11^Department of Paediatrics and Child Health, University of Pretoria, Pretoria, South Africa; ^12^Department of Paediatrics and Child Health, School of Clinical Medicine, College of Health Sciences, University of KwaZulu Natal, Durban, South Africa

**Keywords:** bronchiectasis, children, Africa, chronic cough, HIV

## Abstract

Bronchiectasis (BE) is a chronic condition affecting the bronchial tree. It is characterized by the dilatation of large and medium-sized airways, secondary to damage of the underlying bronchial wall structural elements and accompanied by the clinical picture of recurrent or persistent cough. Despite an increased awareness of childhood BE, there is still a paucity of data on the epidemiology, pathophysiological phenotypes, diagnosis, management, and outcomes in Africa where the prevalence is mostly unmeasured, and likely to be higher than high-income countries. Diagnostic pathways and management principles have largely been extrapolated from approaches in adults and children in high-income countries or from data in children with cystic fibrosis. Here we provide an overview of pediatric BE in Africa, highlighting risk factors, diagnostic and management challenges, need for a global approach to addressing key research gaps, and recommendations for practitioners working in Africa.

## Introduction

Bronchiectasis (BE) is a chronic condition affecting the bronchial tree, characterized by the dilatation of large and medium-sized airways secondary to damage of the underlying bronchial wall structural elements (1). The European Respiratory Society (ERS) defines childhood BE as “an umbrella term for a clinical syndrome of recurrent or persistent wet/productive cough, airway infection and inflammation, and abnormal bronchial dilatation on chest computed tomography (CT) scans” (2). The condition is therefore an end-point of chronic inflammation from severe or recurrent insults, and an important cause of respiratory disease in children (3). Despite an increased awareness of childhood BE, there is still a paucity of data on the epidemiology, pathophysiological phenotypes, diagnosis, management, and outcomes in Africa where disease burden is likely to be higher than in high-income (HIC) settings. Although over 50% of the world's children live on the African continent, where infectious risk factors for BE are common, there is a gross paucity of relevant data from this region to inform prevention and management practices. Diagnostic pathways and management principles have largely been extrapolated from approaches in adults and children in HIC or from literature on cystic fibrosis (CF). This commentary provides an overview of pediatric BE in Africa, highlighting risk factors for BE, diagnostic and management challenges, the need for a global approach addressing key research gaps, and recommendations for practitioners working in Africa.

## Burden of pediatric bronchiectasis

Bronchiectasis has previously been labeled an “orphan” disease, however, with increasing availability of chest CT imaging, a number of studies have shown that this is not true (2–7). Indeed a disproportionately high incidence of pediatric BE is reported in socioeconomically deprived first nation populations in HIC, including Australia (8), New Zealand (9), Alaska (10) and Canada (11); however BE is by no means restricted to these populations. Documented risk factors for pediatric BE in these settings include: recurrent lower respiratory tract infections (LRTI), pulmonary tuberculosis (PTB), differences in the airway microbiome, genetic predisposition, socioeconomic strata, and poor access to medical care (8, 12–14).

Data from the African continent on the burden of pediatric BE is lacking, however few studies have reported that a high proportion of children attending respiratory and human immunodeficiency virus (HIV) treatment clinics in Africa have BE (15–17). Some factors associated with an increased risk of BE in Africa include: (i) an increased burden of LRTI and severe LRTI, including PTB (18–20), (ii) a heavy burden of pediatric HIV infection, with 60% of the world's children living with HIV (CLWH) residing in sub-Saharan Africa (SSA) (21), (iii) lower childhood vaccine coverage rates for organisms such as *Bordetella pertussis, Streptococcus pneumoniae, Haemophilus influenzae*, and measles; with the African vaccine coverage rates at approximately 79 and 72% for the third diphtheria-tetanus toxoids-pertussis and *Haemophilus influenza* type b vaccines, respectively, 68% for the third dose of pneumococcal conjugate vaccine, and only 36% for the second dose of the measles vaccine (22), (iv) lower socioeconomic status, (v) poor access to medical treatment and medical follow-up (23), and (vi) lower levels of education regarding the identification and management of chronic wet cough and persistent bacterial bronchitis (PBB) in caregivers and medical practitioners (24).

## Pathophysiology

Chronic neutrophilic inflammation promotes damage to mucosal, submucosal, and muscular components of the airway bronchial wall, eventually causing dilatation of the airways. Described as Cole's vicious cycle hypothesis; the chronic inflammation, hypersecretion of mucus, impairment of muco-ciliary clearance mechanisms and bacterial colonization perpetuate the cycle of damage to the airways (25). Recently this process has been defined as a vortex, or the vicious infection-inflammation cyclic theory, where airway dysfunction, inflammatory responses, structural disease, and infection all contribute to development of BE, with a complex interaction between the pathophysiological mechanisms (26). For example, in CLWH, there is not only neutrophilic-driven airway inflammation, but also an exaggerated local and systemic immunological response to bacterial and fungal pathogens, contributing to BE (27). More research is needed in African populations where BE is more prevalent to delineate specific pathophysiological mechanisms to identify pathways for therapeutic intervention.

## Etiology

The etiology of BE is well described in HICs, but the proportional weighting of causes differs between and within populations in the same country or region (6, 11, 13, 28). The largest proportion of pediatric BE, from both HICs and low- and middle-income countries (LMICs), is due to either post-infectious or idiopathic causes (9, 29–31). Other causes include CF, primary ciliary dyskinesia (PCD), primary immunodeficiencies (PID) and aspiration syndromes (29, 30). CF is reported to be more common in children with European ancestry (32), and aspiration syndromes in children with neurological impairment. Data from LMICs such as India and Thailand found that post-infectious causes were the most commonly described; in China, idiopathic and post-infectious causes were most common (13). In less affluent indigenous populations residing in HICs, post-infectious causes are most commonly described, although genetic predisposition may play a role (10, 12, 33). Access to specialized diagnostic tools may also contribute to the changing etiology recently described in Turkey, where improved investigation of BE have led to an increased diagnosis of PCD and PID, and less cases attributed to post-infections causes (34). Describing the etiology of BE through population based studies in Africa is crucial to establishing preventative and management guidelines.

There is also variability with regards to the causative pathogens in post-infectious BE, with PTB being highly prevalent in SSA. In 2020, according to the World Health Organization Global tuberculosis report, Africa accounted for 25% of all new TB cases globally, 12%, of these cases occurring in children aged <15 years, which translates into an estimated 355 000 (uncertainty level 308 000 – 401 000) new cases in children aged <15 years, and 93 000 deaths (35). There is very little data on the characterization of post-TB lung disease, particularly in children, as most TB programs regard TB cure as an end-point to care without long-term follow up (36–38). Lower respiratory tract (including pneumonia) infection is also a risk factor: Western and Central Africa has one of the highest globally reported incidences of pneumonia in children (1 620 per 100 000 children); in a well vaccinated cohort of children from Cape Town, the incidence of LTRTI in the first 2 years of life was reported as 0.51 and 0.25 episodes per child year, respectively (39, 40). With limited molecular characterization of bacterial and viral causes of severe LRTI, risk-stratification of those with severe infections, who may require follow-up, for example with severe adenoviral or B. pertussis infections, is not possible in SSA (41).

More than 67% of the 37.7 million people living with HIV, of which 1.7 million are aged <15 years, reside in SSA. Of these 1.7 million CLWH, only 59% have been initiated on highly active antiretroviral therapy (42). The lungs are a primary target for infections in CLWH, with recurrent and severe LRTI, increased risk of TB infection, infection with opportunistic organisms, as well as immune dysregulation by HIV causing inflammation and an altered lung microbiome, all potential precursors of BE (21, 27, 43–46). In older children and adolescents with perinatally-acquired HIV in Zimbabwe, chronic lung disease was common (86%); between 33% (28/84) and 43% (24/56) had confirmed BE on high-resolution CT (15, 16). Furthermore, prior severe LRTI and PTB were associated with four- to five-fold increased risk of BE respectively in this group, who were well controlled on anti-retroviral therapy (ART) (47). Early access to ART decreases risk of LRTI (48), improves lung function outcomes, and reduces sputum bacteriological carriage in CLWH (49, 50), highlighting the importance of strengthening access to timely HIV diagnosis and prompt ART initiation for all CLWH.

Access to specialized diagnostic tools may also contribute to a change in the etiology. In most African countries, limited financial resources preclude access to extensive investigations for confirming the cause of BE, for example; for CF sweat testing and genetic testing are limited to a few specialized centers in Africa, and local mutation data is largely unavailable. For PCD there is limited testing capacity with ciliary brushings and electron microscopy, as well as genetic testing and exhaled nitric oxide equipment (51); for primary immunodeficiencies (PID) an inability to perform an expensive panel of tests; and a lack of radiology facilities to diagnose aspiration syndromes. The implications of this would be that the underlying diagnosis of the etiology leading to pediatric BE in Africa will remain largely unknown, and the post-infectious and idiopathic groups dominant, until further well-resourced research can be performed in African populations with the full spectrum of diagnostic tools available.

## Diagnosis/investigations

**Figure 1:**
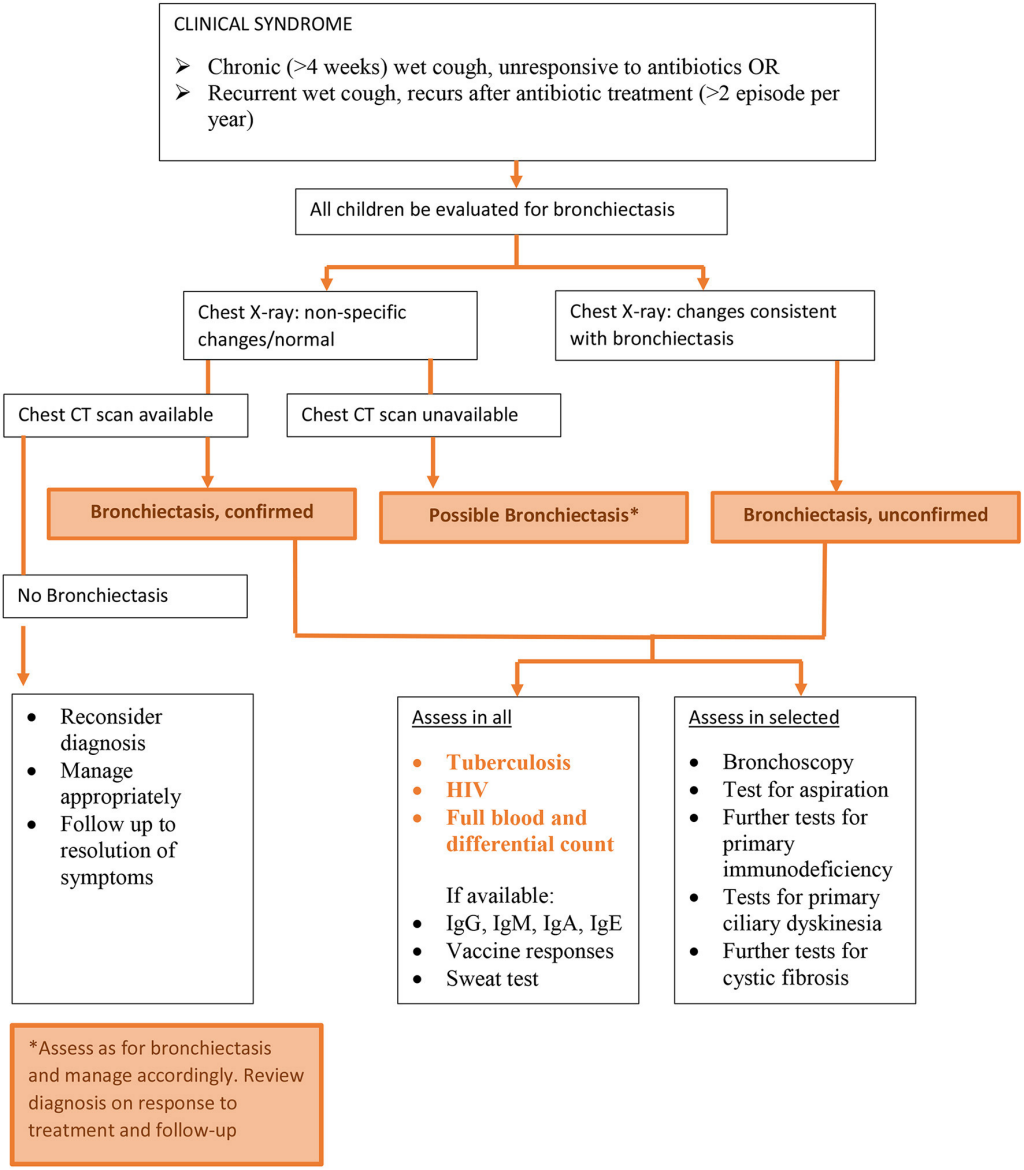
Diagnosing and investigating childhood bronchiectasis in Africa.

According to recent consensus guidelines, the diagnosis of pediatric BE relies on both a consistent clinical syndrome and documented airway dilatation on chest CT scan (2). In Africa, where resources are not readily available or limited to specialized centers, the absence of chest CT confirmation leads to a under-recognition of BE with delays in management and poorer long-term outcomes (15, 52, 53). This lends itself to a lack of public awareness and subsequent decrease in public spending where resources are already in short supply. The chest radiograph may be the only available imaging modality, and is useful for diagnosing the more severe spectrum of BE. It is not uncommon to have non-specific x-ray changes on chest radiography in patients with CT scan confirmed BE, however if specific chest radiography changes are present, it usually indicates severe disease. Therefore, in Africa, an approach to the diagnosis and early implementation of treatment for BE may need to be based on clinical and chest radiograph criteria that is accessible and feasible–we propose an alternative diagnostic algorithm (Figure 1).

It has been suggested that a minimum panel of tests should be performed in children with confirmed BE (2). These include a full blood count, immunological testing (total Immunoglobulins A, G, M and E and specific immunoglobulin vaccine responses), a sweat test, spirometry, and lower airway bacteriological surveillance. Additional tests are recommended based on the clinical presentation of the child, and these may include more substantive immunological tests, bronchoscopy for airway bacteriology, and tests for the work-up of aspiration and gastro-esophageal reflux disease, as well as for PCD. These minimum panel of tests is however based on available data from HIC, and it remains unknown whether this panel would comprehensively cover the main etiologies from Africa, and is definitely not economically viable for most of the continent. The guidelines do make provision for additional testing, including HIV testing and PTB screening, in areas where these diseases are endemic, such as in SSA.

HIV testing in SSA is routinely and vigorously applied, leading to the earlier diagnosis and management and a subsequent decrease disease burden. Further, work-up for PTB is extensively performed for children with LRTI. These approaches may contribute to the decreasing the burden of pediatric BE, although studies confirming this need to be performed.

Sputum surveillance is one of the cornerstones in the work-up for pediatric BE, and provides objective data on the real-time management of an acute exacerbations in BE. There are a few studies describing airway bacteriology from the African continent, and the results are similar to those undertaken outside Africa (43, 54). However, a higher carriage rate of *Moraxella catarrhalis* and *S. pneumonia* (including higher rates of penicillin non-susceptibility) has been reported in CLWH that have chronic lung disease (CLD) compared to those without CLD (50).

Pulmonary function testing is not readily available in SSA, except in a few specialized centers, severely impacting the ability to assess the severity of BE or to gauge the response to treatment. Recent efforts to collect normal pulmonary function data in Africa has gained momentum, and recommendations for pulmonary function equations to be used in Africa has been published (55–59). Pulmonary function data on HIV-exposed uninfected children and CLWH have been published (15, 60–62). Further studies from Africa describing pulmonary function data in pediatric BE, and stratifying according to etiology, is urgently required.

There is a need to develop a diagnostic approach algorithm that is suited to Africa, and based on data generated from local research complementing data extrapolated from HICs, thereby taking into consideration the economic and logistical constraints found in Africa, but also optimizing the utility of the resources that are available.

## Management

Basic management principles of BE include: treating the underlying cause that has resulted in the BE, managing the symptoms and complications, and protecting the lungs from further injury. Lung protective strategies include aggressive treatment of any future LRTI or respiratory exacerbations, immunization against common childhood respiratory pathogens, such *as* Influenza, *S. pneumonia, H. influenzae, B. pertussis*, and measles, and the avoidance of environmental tobacco smoke, including second and third-hand smoking, and indoor biomass fuel exposure. Specific treatment should be tailored to any underlying condition and appropriate management instituted, we suggest a panel of management strategies stratified to availability and level of care in Africa (Table 1).

**Table 1:** Management of bronchiectasis in Africa.

**Type of therapy** ^a^	**Drug**	**Level of care**
Airway clearance techniques	Airway clearance therapy	Primary
Antibiotics for exacerbations^b^	Amoxycillin-clavulanate	Primary
Bronchodilators	Inhaled salbutamol^c^	Primary
Immunomodulators^d^	Azithromycin	Secondary to tertiary
	*Alternative*	
	Erythromycin	
Mucoactive agents	Hypertonic saline^b^	Secondary
Pseudomonas eradication	Ciprofloxacillin	Tertiary outpatient
Vaccination^e^	Influenza vaccine yearly	Secondary or Tertiary

a Green: Essential and currently largely available at primary health care level; Orange: Secondary level care and desirable; Red: Largely available only at tertiary and quaternary level of care.

b Secondary or tertiary level therapy if infections are persistent or recurrent, where antibiotic choice guided by sputum sample microscopy, culture and sensitivity.

c For use prior to hypertonic saline if bronchoconstriction present and in case of frequent exacerbations.

d If > 3 exacerbations per year and hospitalization in last 3 months OR proven Pseudomonas aeruginosa infection, based on Serisier DJ, Martin ML, McGuckin MA, Lourie R, Chen AC, Brain B et al. Effect of Long-term, Low-Dose Erythromycin on Pulmonary Exacerbations Among Patients With Non-Cystic Fibrosis Bronchiectasis: The BLESS Randomized Controlled Trial. JAMA. 2013;309(12):1260-1267. doi: 10.1001/jama.2013.2290 and Kapur N, Stroil-Salama E, Morgan L, Yerkovich S, Holmes-Liew CL, King P et al. Factors associated with “Frequent Exacerbator” phenotype in children with bronchiectasis: The first report on children from the Australian Bronchiectasis Registry. Respir Med. 2021 Nov;188:106627. doi: 10.1016/j.rmed.2021.106627. Epub 2021 Sep 25. PMID: 34592538.

e This refers to the annual influenza virus vaccination only, and not the standard childhood immunisations.

The backbone of treatment for BE is airway clearance and treatment of acute infections or exacerbations (2). Airway clearance is achieved through a wide variety of techniques, from positioning and chest percussion alone, to active cycle breathing techniques, and the application of instruments to aid in airway clearance. The manual airway clearance techniques are freely available to all and easy to learn and should be actively taught and encouraged in countries where instruments used for airway clearance techniques are not easily available or affordable (63, 64). Institution of low-cost pulmonary rehabilitation (PR) programs can be done for children to improve quality of life and even in remote rural areas, use of mobile technology can assist parents and children with PR.

LRTI episodes and severity can effectively be reduced with the appropriate use of affordable antibiotics and increasing immunization coverage, decreasing both new cases of post-infectious BE, as well as preventing further damage to the lungs of children with BE (65, 66). In Africa, there is the potential to greatly decrease the burden of post-infectious BE with early identification of children at risk of developing BE. Appropriate antibiotic therapy and follow-up of children with the chronic “wet” cough are key interventions. Recent studies have shown that chronic “wet cough” may be part of a spectrum that encompasses chronic “wet” cough, PBB, and eventually BE, and that a window of opportunity is afforded to clinicians to arrest the development of BE if managed timeously and correctly (67). Early recognition by the caregiver and medical practitioner is therefore of paramount importance. There has been an increase in education regarding these conditions in HICs recently, and this has also started expanding to Africa, although much work remains (68, 69).

General alleviation of poverty, with improved access to medical care and medical education will decrease the burden of LRTI in the community, as well as facilitate early diagnosis and timely referral of children with BE to specialized centers. Although it is not possible to access or afford many of the specialized treatments targeting specific etiologies of BE in Africa, such as CF or PID, treatment options are available to manage some of the most common causes of BE in these areas. Advocacy is required to create regional or national centers of excellence to better understand the etiology and outcome of BE in Africa, and more importantly, to address inequalities in child health in Africa and globally.

## Outcome

The outcomes of children with BE are dependent on both the underlying disease leading to the BE, as well as the severity of the BE itself (70). Children with BE that is not due to CF tend to have worse pulmonary function than those with CF (71). Conflicting results have been reported on the long-term lung function trajectories of children in Africa, with the outcome or economic burden of BE in Africa largely unknown. Early HAART initiation has been associated with lung function improvement in the first 2 years following initiation, but this may not be sustained; the impact of nutrition on BE and lung function trajectories, needs further exploration, as severe acute malnutrition was not associated with worse lung function at seven years post-treatment, in survivors, however, this was not in children with BE (49, 72). Longitudinal data needs to be collected on children with BE from Africa, to document the effect of differing etiologies of bronchiectasis on the long-term outcome of patients. Studies from HICs indicate that there is an increased rate of health care utilization among children with BE (7, 49, 52, 72, 73).

## Research

The establishment of registries are key to answer many key questions including outcomes of children with BE. Until recently there have been no known registries or databases from Africa, but in 2020 the BACPAC (Bronchiectasis in African Children: Prevalence, Etiology and clinical spectrum) Network was established in South Africa, with the potential to expand through-out Africa. This network has two objectives, the first being to establish a bronchiectasis registry, and the second to investigate children with chronic (>8 weeks) cough, who do not respond to appropriate antibiotic treatment. This network will provide some of the first descriptive data on children with bronchiectasis from a developing country, thereby providing population specific and LMIC information that has been lacking so far in the study of pediatric bronchiectasis.

## Recommendations

As highlighted above, there are many challenges in all aspects of BE care in Africa, including early case detection, diagnosis, and management. Large strides have been made in the past decade with African-led research examining important aspects in pulmonary health, however efforts to address the upstream factors that continue to drive child health inequities need to be addressed. In Africa, large improvements can be achieved even within the financial and logistical constraints that are prevalent in Africa. These can be broadly divided into improving current definitions of bronchiectasis (to facilitate appropriate treatment and address knowledge gaps), contextual diagnostic and management pathways, establishing disease registries, strengthening research capacity and collaborations, and advocacy.

### Definition

We propose an alternative definition of BE that is focused on prevention and clinical management, can be applied more easily in Africa and other LMICs, and will help facilitate global efforts to address knowledge gaps. This definition is based primarily on the presence of the clinical syndrome of BE and chest radiograph, which is much more readily available and reduces the need for CT scan to select cases, whilst offering pathways to care when CT scans are not accessible. The proposed new definition would be divided into three groups (Suggested definition and flow detailed in Figure 1):

i) Confirmed BE: clinical syndrome of BE and CT scan confirmation of dilated bronchi.
ii) Unconfirmed BE: clinical syndrome of BE and chest x-ray changes consistent with BE (not confirmed on CT).
iii) Possible BE: clinical syndrome of BE, but no chest x-ray evidence of BE nor access to CT scan.

### Investigation and treatment

Children with possible BE should be investigated for etiology and baseline severity as with unconfirmed/confirmed BE. All possible / unconfirmed / confirmed BE should be started on a pulmonary rehabilitation (PR) program taking into context available resources and education of caregivers and children. Children with possible BE require regular follow up at least 3–6 monthly and a clear written plan to the primary care center/clinic in case of acute exacerbations, particularly to those living in more remote rural areas. Harm reduction with education of caregivers and children on the exposures to tobacco smoke and biomass fuel should form part of the package of care.

Contextual African diagnostic and management pathways need to be established, based on local population data and take into consideration the economic and logistical constraints, as well as focus on African etiology and management options. These pathways need to be widely distributed and available to all health care workers, and in due time become standard practice of care. In this way early detection and management of BE will improve the long-term lung health on the continent.

### Research capacity strengthening

A Pan-African Research collaboration network needs to be established to better define chronic respiratory disease including BE. The Pan African Thoracic Society would be a platform to facilitate and create such a collaboration. Well-designed African-led studies that would address the research gaps and inform policy to improve outcomes are needed. Trans-continental collaboration with other established networks and research collaborators in HIC are also necessary to share experiences and expertise.

### Registries

The expansion of the existing BACPAC registry to a Pan African Registry. Representation from the BACPAC group has been included in the Children's Bronchiectasis Education, Advocacy and Research Network (Child-Bear-Net), a group of doctors from around the globe, with a joint interest of setting up regional and global bronchiectasis registries, in order to adequately describe the prevalence of the problem and to improve the general care and management, and thereby the outcomes of children with bronchiectasis, as well as to promote parental and caregiver education regarding the disease. Consideration for including updated definitions in this registry is recommended.

### Advocacy

In the meantime, while we address the unmet needs in BE in Africa, much can be done to improve the general management and therefore the outlook of children with BE in Africa. Community and health care worker education and general lung protective measures can be strengthened in the community, through improved access to immunization programs, prevention and early diagnosis of HIV, and access to antibiotics for LRTI, including PTB treatment, as well as improved access to ARVs. There is a need to train and improve awareness among clinicians on existence of BE in children, identification of at risk children, and early recognition of clinical signs suggestive of BE.

## Conclusion

Due to limited resources and access to special investigations BE is largely under-recognized and under-appreciated in Africa. Providing simple algorithms to identify at risk children may assist in earlier identification and management of children, hence preventing or ameliorating disease. Strengthening research capacity through pan-African research collaborative networks and creation of a registry will assist in answering key research gaps and informing optimal clinical practice and effective policy.

## Author contributions

CV, DG, AG, and RM conceptualized and designed the study. All authors reviewed, revised, and approved the final manuscript as submitted.

## Conflict of interest

The authors declare that the research was conducted in the absence of any commercial or financial relationships that could be construed as a potential conflict of interest.

## Publisher's note

All claims expressed in this article are solely those of the authors and do not necessarily represent those of their affiliated organizations, or those of the publisher, the editors and the reviewers. Any product that may be evaluated in this article, or claim that may be made by its manufacturer, is not guaranteed or endorsed by the publisher.

## References

[1] Field CE. Bronchiectasis in childhood; aetiology and pathogenesis, including a survey of 272 cases of doubtful irreversible bronchiectasis. Pediatrics. (1949) 4:231–48. 10.1542/peds.4.2.231 18137846

[2] Chang AB Fortescue R Grimwood K Alexopoulou E. European respiratory society guidelines for the management of children and adolescents with bronchiectasis. Eur Respir J. (2021) 58:2002990. 10.1183/13993003.02990-2020 33542057

[3] Chalmers JD. Bronchiectasis from 2012 to 2022. Clin Chest Med. (2022) 43:1–6. 10.1016/j.ccm.2021.12.001 35236552

[4] Chang AB Bush A Grimwood K. Bronchiectasis in children: diagnosis and treatment. Lancet. (2018) 392:866–79. 10.1016/S0140-6736(18)31554-X 30215382

[5] Goyal V Grimwood K Marchant J Masters IB Chang AB. Pediatric bronchiectasis: no longer an orphan disease. Pediatr Pulmonol. (2016) 51:450–69. 10.1002/ppul.23380 26840008

[6] Monteagudo M Rodríguez-Blanco T Barrecheguren M Simonet P Miravitlles M. Prevalence and incidence of bronchiectasis in Catalonia, Spain: a population-based study. Respir Med. (2016) 121:26–31. 10.1016/j.rmed.2016.10.014 27888988

[7] Quint JK Smith MP. Paediatric and adult bronchiectasis: Diagnosis, disease burden and prognosis. Respirology. (2019) 24:413–22. 10.1111/resp.13495 30779274

[8] O'Grady KA Torzillo PJ Chang AB. Hospitalisation of Indigenous children in the Northern Territory for lower respiratory illness in the first year of life. Med J Aust. (2010) 192:586–90. 10.5694/j.1326-5377.2010.tb03643.x 20477735

[9] Edwards EA Asher MI Byrnes CA. Paediatric bronchiectasis in the twenty-first century: experience of a tertiary children's hospital in New Zealand. J Paediatr Child Health. (2003) 39:111–7. 10.1046/j.1440-1754.2003.00101.x 12603799

[10] Singleton R Morris A Redding G Poll J Holck P Martinez P. Bronchiectasis in Alaska Native children: causes and clinical courses. Pediatr Pulmonol. (2000) 29:182–7. 10.1002/(sici)1099-0496(200003)29:3<182::aid-ppul5>3.0.co;2-t 10686038

[11] Das L Kovesi TA. Bronchiectasis in children from Qikiqtani (Baffin) Region, Nunavut, Canada. Ann Am Thorac Soc. (2015) 12:96–100. 10.1513/AnnalsATS.201406-257OC 25496305

[12] de Boer S Lewis CA. Ethnicity, socioeconomic status and the severity and course of non-cystic fibrosis bronchiectasis. Int Med J. (2018) 48:845–50. 10.1111/imj.13739 29345411

[13] Chandrasekaran R Mac Aogáin M Chalmers JD Elborn SJ Chotirmall SH. Geographic variation in the aetiology, epidemiology and microbiology of bronchiectasis. BMC Pulm Med. (2018) 18:83. 10.1186/s12890-018-0638-0 29788932 PMC5964678

[14] Singleton RJ Valery PC Morris P Byrnes CA Grimwood K Redding G. Indigenous children from three countries with non-cystic fibrosis chronic suppurative lung disease/bronchiectasis. Pediatr Pulmonol. (2014) 49:189–200. 10.1002/ppul.22763 23401398

[15] Ferrand RA Desai SR Hopkins C Elston CM Copley SJ Nathoo K. Chronic lung disease in adolescents with delayed diagnosis of vertically acquired HIV infection. Clin Infect Dis. (2012) 55:145–52. 10.1093/cid/cis271 22474177 PMC3369563

[16] Desai SR Nair A Rylance J Mujuru H Nathoo K McHugh G. Human immunodeficiency virus-associated chronic lung disease in children and adolescents in Zimbabwe: chest radiographic and high-resolution computed tomographic findings. Pediatr Pulmonol. (2018) 66:274–81. 10.1093/cid/cix778 29020237 PMC5850005

[17] du Plessis AM Andronikou S Zar HJ. Chest imaging findings of chronic respiratory disease in HIV-infected adolescents on combined anti retro viral therapy. Paediatr Respir Rev. (2021) 38:16–23. 10.1016/j.prrv.2020.06.022 33139219

[18] Levels and Trends in Child Mortality. Report 2020, Estimates Developed by the United Nations Inter-Agency Group for Child Mortality Estimation. New York, NY: United Nations Children's Fund (2020).

[19] World Health Statistics 2019. Monitoring Health for the SDGs, Sustainable Development Goals. Geneva: World Health Organization (2019) Contract No.: Licence: CC BY-NC-SA 3.0 IGO.

[20] Yerramsetti S Cohen T Atun R Menzies NA. Global estimates of paediatric tuberculosis incidence in 2013-19: a mathematical modelling analysis. Lancet Glob Health. (2022) 10:e207–e15. 10.1016/S2214-109X(21)00462-9 34895517 PMC8800006

[21] UNAIDS Data 2019 (2019). Available online at: https://www.unaids.org/en/resources/documents/2019/2019-UNAIDS-data

[22] Muhoza P Danovaro-Holliday MC Diallo MS Murphy P Sodha SV Requejo JH. Routine vaccination coverage - worldwide, 2020. MMWR Morbid Mortal Wkly Rep. (2021) 70:1495–500. 10.15585/mmwr.mm7043a1 34710074 PMC8553029

[23] Brakema EA Tabyshova A van der Kleij R Sooronbaev T Lionis C Anastasaki M. The socioeconomic burden of chronic lung disease in low-resource settings across the globe - an observational FRESH AIR study. Respir Res. (2019) 20:291. 10.1186/s12931-019-1255-z 31864411 PMC6925865

[24] Laird P Walker R Lane M Chang AB Schultz A. We won't find what we don't look for: Identifying barriers and enablers of chronic wet cough in Aboriginal children. Respirology. (2020) 25:383–92. 10.1111/resp.13642 31344317

[25] Cole PJ. Inflammation: a two-edged sword–the model of bronchiectasis. Eur J Respir Dis Suppl. (1986) 147:6–15. 3533593

[26] Flume PA Chalmers JD Olivier KN. Advances in bronchiectasis: endotyping, genetics, microbiome, and disease heterogeneity. Lancet. (2018) 392:880–90. 10.1016/S0140-6736(18)31767-7 30215383 PMC6173801

[27] Masekela R Anderson R Moodley T Kitchin OP Risenga SM Becker PJ. HIV-related bronchiectasis in children: an emerging spectre in high tuberculosis burden areas. Int J Tuberc Lung Dis. (2012) 16:114–9. 10.5588/ijtld.11.0244 22236856

[28] Chang AB Grimwood K Mulholland EK Torzillo PJ. Bronchiectasis in indigenous children in remote Australian communities. Med J Aust. (2002) 177:200–4. 10.5694/j.1326-5377.2002.tb04733.x 12175325

[29] Satirer O Mete Yesil A Emiralioglu N Tugcu GD Yalcin E Dogru D. A review of the etiology and clinical presentation of non-cystic fibrosis bronchiectasis: a tertiary care experience. Respir Med. (2018) 137:35–9. 10.1016/j.rmed.2018.02.013 29605210

[30] Lee E Shim JY Kim HY Suh DI Choi YJ Han MY. Clinical characteristics and etiologies of bronchiectasis in Korean children: a multicenter retrospective study. Respir Med. (2019) 150:8–14. 10.1016/j.rmed.2019.01.018 30961955

[31] McCallum GB Binks MJ. The epidemiology of chronic suppurative lung disease and bronchiectasis in children and adolescents. Front Pediatr. (2017) 5:27. 10.3389/fped.2017.00027 28265556 PMC5316980

[32] Scotet V L'Hostis C. The changing epidemiology of cystic fibrosis: incidence, survival and impact of the CFTR gene discovery. Genes. (2020) 11:589. 10.3390/genes11060589 32466381 PMC7348877

[33] Chang AB Grimwood K Maguire G King PT Morris PS Torzillo PJ. Management of bronchiectasis and chronic suppurative lung disease in indigenous children and adults from rural and remote Australian communities. Med J Aust. (2008) 189:386–93. 10.5694/j.1326-5377.2008.tb02085.x 18837683

[34] Eralp EE Gokdemir Y Atag E Ikizoglu NB Ergenekon P Yegit CY. Changing clinical characteristics of non-cystic fibrosis bronchiectasis in children. BMC Pulm Med. (2020) 20:172. 10.1186/s12890-020-01214-7 32546272 PMC7298950

[35] Global Tuberculosis Report 2020. Geneva: World Health Organization (2020). Licence: CC BY-NC-SA 3.0 IGO. Contract No.: Licence: CC BY-NC-SA 3.0 IGO.

[36] Allwood BW Myer L Bateman ED. A systematic review of the association between pulmonary tuberculosis and the development of chronic airflow obstruction in adults. Respiration. (2013) 86:76–85. 10.1159/000350917 23652030

[37] Meghji J Lesosky M. Patient outcomes associated with post-tuberculosis lung damage in Malawi: a prospective cohort study. Respir Infect. (2020) 75:269–78. 10.1136/thoraxjnl-2019-213808 32102951 PMC7063395

[38] Allwood BW Byrne A Meghji J Rachow A van der Zalm MM Schoch OD. Post-tuberculosis lung disease: clinical review of an under-recognised global challenge. Respiration. (2021) 100:751–63. 10.1159/000512531 33401266

[39] Pneumonia 2022. Available online at: https://data.unicef.org/topic/child-health/pneumonia/ (accessed June 22, 2022).

[40] Roux DM Nicol MP Myer L Vanker A Stadler JAM von Delft E. Lower respiratory tract infections in children in a well-vaccinated South African birth cohort: spectrum of disease and risk factors. Clin Infect Dis. (2019) 69:1588–96. 10.1093/cid/ciz017 30925191

[41] Nicholson DP. Pulmonary collapse in pertussis. Arch Dis Child. (1949) 24:29–40. 10.1136/adc.24.117.29 18114485 PMC1988205

[42] Geneva: Joint United Nations Progamme on HIV/AIDS. Geneva: (2021). Contract No.: Licence: CC BY-NC-SA 3.0 IGO.

[43] Masekela R Vosloo S Venter SN de Beer WZ Green RJ. The lung microbiome in children with HIV-bronchiectasis: a cross-sectional pilot study. BMC Pulm Med. (2018) 18:87. 10.1186/s12890-018-0632-6 29788934 PMC5964725

[44] Theodoratou E McAllister DA Reed C Adeloye DO Rudan I Muhe LM. Global, regional, and national estimates of pneumonia burden in HIV-infected children in 2010: a meta-analysis and modelling study. Lancet Infect Dis. (2014) 14:1250–8. 10.1016/S1473-3099(14)70990-9 25455992 PMC4242006

[45] Fry SHL Barnabas SL Cotton MF. Tuberculosis and HIV—an update on the “cursed duet” in children. Front Pediatr. (2019) 7:159. 10.3389/fped.2019.00159 32211351 PMC7073470

[46] Githinji L Zar HJ. Respiratory complications in children and adolescents with human immunodeficiency virus. Pediatr Clin North Am. (2021) 68:131–45. 10.1016/j.pcl.2020.09.016 33228928

[47] du Plessis AM Andronikou S Machemedze T Griffith-Richards S Myer L Mahtab S. High-resolution computed tomography features of lung disease in perinatally HIV-infected adolescents on combined antiretroviral therapy. Pediatr Pulmonol. (2019) 54:1765–73. 10.1002/ppul.24450 31338996

[48] de Campos KR Granga DD Olorunju S Masekela R. The impact of highly active antiretroviral therapy on the burden of bacterial lower respiratory tract infections in children. S Afr Med J. (2015) 105:554–7. 10.7196/SAMJnew.7820 26428750

[49] Rylance S Rylance J McHugh G Majonga E Bandason T Mujuru H. Effect of antiretroviral therapy on longitudinal lung function trends in older children and adolescents with HIV-infection. PLoS ONE. (2019) 14:e0213556. 10.1371/journal.pone.0213556 30897116 PMC6428265

[50] Abotsi RE Nicol MP McHugh G Simms V Rehman AM Barthus C. Prevalence and antimicrobial resistance profiles of respiratory microbial flora in African children with HIV-associated chronic lung disease. BMC Infect Dis. (2021) 21:216. 10.1186/s12879-021-05904-3 33632144 PMC7908671

[51] Shapiro AJ Zariwala MA Ferkol T Davis SD Sagel SD Dell SD. Diagnosis, monitoring, and treatment of primary ciliary dyskinesia: PCD foundation consensus recommendations based on state of the art review. Pediatr Pulmonol. (2016) 51:115–32. 10.1002/ppul.23304 26418604 PMC4912005

[52] Meghji J Mortimer K Agusti A Allwood BW Asher I Bateman ED. Improving lung health in low-income and middle-income countries: from challenges to solutions. Lancet. (2021) 397:928–40. 10.1016/S0140-6736(21)00458-X 33631128

[53] Kapur N Masters IB Chang AB. Longitudinal growth and lung function in pediatric non-cystic fibrosis bronchiectasis: what influences lung function stability? Chest. (2010) 138:158–64. 10.1378/chest.09-2932 20173055

[54] Verwey C Velaphi S Khan R. Bacteria isolated from the airways of paediatric patients with bronchiectasis according to HIV status. S Afr Med J. (2017) 107:435–9. 10.7196/SAMJ.2017.v107i5.10692 28492126

[55] Masekela R Koegelenberg CFN Gray DM. Guidance to the applicability of the global lung initiative spirometry reference equations for South African populations. S Afr Med J. (2020) 111:13186. 10.7196/SAMJ.2021.v111i2.15439 33334388

[56] Masekela R Hall GL Stanojevic S Sartorius B MacGinty R Saad HB. An urgent need for African spirometry reference equations: the paediatric and adult African spirometry study. Int J Tuberc Lung Dis. (2019) 23:952–8. 10.5588/ijtld.18.0442 31533886

[57] Smith SJ Gray DM MacGinty RP Hall GL. Choosing the better global lung initiative 2012 equation in South African population groups. Am J Respir Crit Care Med. (2020) 202:1724–7. 10.1164/rccm.202005-2085LE 32757942 PMC7737576

[58] Madanhire T Ferrand RA Attia EF Sibanda EN Rusakaniko S Rehman AM. Validation of the global lung initiative 2012 multi-ethnic spirometric reference equations in healthy urban Zimbabwean 7-13 year-old school children: a cross-sectional observational study. BMC Pulm Med. (2020) 20:56. 10.1186/s12890-020-1091-4 32111226 PMC7048020

[59] Pefura-Yone EW Balkissou AD Poka-Mayap V Djenabou A Massongo M Ofimboudem NA. Spirometric reference equations for cameroonians aged 4 to 89 years derived using lambda, mu, sigma (LMS) method. BMC Pulm Med. (2021) 21:344. 10.1186/s12890-021-01705-1 34732174 PMC8565080

[60] Gray DM Wedderburn CJ MacGinty RP McMillan L Jacobs C Stadler JAM. Impact of HIV and antiretroviral drug exposure on lung growth and function over 2 years in an African Birth Cohort. AIDS. (2020) 34:549–58. 10.1097/QAD.0000000000002444 31714357 PMC7050792

[61] Githinji LN Gray DM Hlengwa S Machemedze T Zar HJ. Longitudinal changes in spirometry in South African adolescents perinatally infected with human immunodeficiency virus who are receiving antiretroviral therapy. Clin Infect Dis. (2020) 70:483–90. 10.1093/cid/ciz255 30938406 PMC7188230

[62] Mwalukomo T Rylance SJ Webb EL Anderson S O'Hare B van Oosterhout JJ. Clinical characteristics and lung function in older children vertically infected with human immunodeficiency virus in Malawi. J Pediatric Infect Dis Soc. (2016) 5:161–9. 10.1093/jpids/piv045 26407277 PMC5407134

[63] Lee AL Burge AT Holland AE. Airway clearance techniques for bronchiectasis. Cochrane database Syst Rev. (2015) 2015:Cd008351. 10.1002/14651858.CD008351.pub3 26591003 PMC7175838

[64] Lee AL Burge AT Holland AE. Positive expiratory pressure therapy versus other airway clearance techniques for bronchiectasis. Cochrane database Syst Rev. (2017) 9:Cd011699. 10.1002/14651858.CD011699.pub2 28952156 PMC6483817

[65] von Gottberg A de Gouveia L Tempia S Quan V Meiring S von Mollendorf C. Effects of vaccination on invasive pneumococcal disease in South Africa. N Engl J Med. (2014) 371:1889–99. 10.1056/NEJMoa1401914 25386897

[66] Zar HJ Moore DP Andronikou S Argent AC Avenant T Cohen C. Diagnosis and management of community-acquired pneumonia in children: South African thoracic society guidelines. Afr J Thorac Crit Care Med. (2020) 26:1–14. 10.7196/AJTCCM.2020.v26i3.104 34471872 PMC7433705

[67] Chang AB Marchant JM. Protracted bacterial bronchitis is a precursor for bronchiectasis in children: myth or maxim? Breathe. (2019) 15:167–70. 10.1183/20734735.0178-2019 31508153 PMC6717611

[68] Laird P Walker R Lane M Totterdell J Chang AB Schultz A. Recognition and management of protracted bacterial bronchitis in Australian aboriginal children: a knowledge translation approach. Chest. (2021) 159:249–58. 10.1016/j.chest.2020.06.073 32673622

[69] Redding G Collaro AJ. Culturally Appropriate outreach specialist respiratory medical care improves the lung function of children in regional and remote Queensland. Int J Circumpolar Health. (2020) 198:361–9. 10.1007/s00408-020-00332-7 32078041

[70] Nathan AM de Bruyne JA Eg KP Thavagnanam S. Review: quality of life in children with non-cystic fibrosis bronchiectasis. Front Pediatr. (2017) 5:84. 10.3389/fped.2017.00084 28596950 PMC5442180

[71] Prentice BJ Wales S Doumit M Owens L. Children with bronchiectasis have poorer lung function than those with cystic fibrosis and do not receive the same standard of care. Pediatr Pulmonol. (2019) 54:1921–6. 10.1002/ppul.24491 31475469

[72] Rylance S Masekela R Banda NPK Mortimer K. Determinants of lung health across the life course in sub-Saharan Africa. Int J Tuberc Lung Dis. (2020) 24:892–901. 10.5588/ijtld.20.0083 33156755

[73] Lovie-Toon YG Grimwood K Byrnes CA Goyal V Busch G Masters IB. Health-resource use and quality of life in children with bronchiectasis: a multi-center pilot cohort study. BMC Health Serv Res. (2019) 19:561. 10.1186/s12913-019-4414-5 31409413 PMC6693266

